# Risk factors for preoperative deep venous thrombosis in hip fracture patients: a meta-analysis

**DOI:** 10.1186/s10195-022-00639-6

**Published:** 2022-04-07

**Authors:** Tao Wang, Junfei Guo, Yubin Long, Yingchao Yin, Zhiyong Hou

**Affiliations:** 1 Department of Orthopaedic Surgery, Third Hospital of Hebei Medical University, Shijiazhuang, Hebei People’s Republic of China; 2 Orthopaedic Research Institute of Hebei Province, Shijiazhuang, Hebei People’s Republic of China; 3 NHC Key Laboratory of Intelligent Orthopaedic Equipment (Third Hospital of Hebei Medical University), Shijiazhuang, China

**Keywords:** Risk factors, Preoperative deep venous thrombosis, Hip fractures, Meta-analysis

## Abstract

**Study design:**

A meta-analysis.

**Background:**

Hip fracture (HF), as common geriatric fracture, is related to increased disability and mortality. Preoperative deep vein thrombosis (DVT) is one of the most common complications in patients with hip fractures, affecting 8–34.9% of hip fracture patients. The study aimed to assess the risk factors of preoperative DVT after hip fractures by meta-analysis.

**Methods:**

An extensive search of the literature was performed in the English databases of PubMed, Embase, and the Cochrane Library; and the Chinese databases of CNKI and WAN FANG. We collected possible predictors of preoperative DVT from included studies, and data analysis was conducted with RevMan 5.3 and STATA 12.0.

**Results:**

A total of 26 English articles were included, and the rate of DVT was 16.6% (1627 of 9823 patients) in our study. Our findings showed that advanced age [*p* = 0.0003, OR = 0.13 95% CI (0.06, 0.21)], female patients [*p* = 0.0009, OR = 0.82 95% CI (0.72, 0.92)], high-energy injury [*p* = 0.009, OR = 0.58 95% CI (0.38, 0.87)], prolonged time from injury to admission [*p* < 0.00001, OR = 0.54 95% CI (0.44, 0.65)], prolonged time from injury to surgery [*p* < 0.00001, OR = 2.06, 95% CI (1.40, 2.72)], hemoglobin [*p* < 0.00001, OR = − 0.32 95% CI (− 0.43, − 0.21)], coronary heart disease [*p* = 0.006, OR = 1.25 95% CI (1.07, 1.47)], dementia [*p* = 0.02, OR = 1.72 95% CI (1.1, 2.67)], liver and kidney diseases [*p* = 0.02, OR = 1.91 95% CI (1.12, 3.25)], pulmonary disease [*p* = 0.02, OR = 1.55 95% CI (1.07, 2.23)], smoking [*p* = 0.007, OR = 1.45 95% CI (1.11, 1.89)], fibrinogen [*p* = 0.0005, OR = 0.20 95% CI (0.09, 0.32)], anti-platelet drug [p = 0.01, OR = 0.51 95% CI (0.30, 0.85)], C-reactive protein [*p* = 0.02, OR = 5.95 95% CI (1.04, 10.85)], < 35 g/l albumin [*p* = 0.006, OR = 1.42 95% CI (1.1, 1.82)], and thrombosis history [*p* < 0.00001, OR = 5.28 95% CI (2.85, 9.78)] were risk factors for preoperative DVT.

**Conclusions:**

Many factors, including advanced age, female patients, high-energy injury, prolonged time from injury to admission, prolonged time from injury to surgery, patients with a history of coronary heart disease, dementia, liver and kidney diseases, pulmonary disease, smoking, and thrombosis, fibrinogen, C-reactive protein, and < 35 g/l albumin, were found to be associated with preoperative DVT. Our findings suggested that the patient with above characteristics might have preoperative DVT.

*Level of evidence:* Level III.

**Supplementary Information:**

The online version contains supplementary material available at 10.1186/s10195-022-00639-6.

## Introduction

Hip fracture (HF), including intertrochanteric fracture, femur neck fracture, and subtrochanteric fracture, as one of the most geriatric fractures associated with osteoporosis [1], is expected to affect about 6 million people by the year 2050 worldwide [2]. Previous studies have demonstrated a close relationship between geriatric hip fractures and perioperative morbidity and mortality at 1 year [3–5]. The treatment of geriatric hip fracture patients is a great challenge due to multiple medical comorbidities and serious perioperative complications [6]. Obtaining stable reduction and fixation to permit early mobilization is critical to decreasing the development of perioperative complications. Early surgery is thought to be the best option for HF patients to reduce the risk of perioperative complications and death [7].

Deep venous thrombosis (DVT), pneumonia, urinary tract infections, sarcopenia, and delirium are common perioperative complications [6, 8, 9]. Both preoperative and postoperative DVT are common conditions in patients with hip fractures due to hypercoagulable states and immobilization [8, 10, 11]. Preoperative DVT, which affects 8–34.9% of hip fracture patients and may be as high as 62% in those with delayed operations [10–12], plays a critical role in HF patients’ preoperative waiting time [13]. Zhao [14] reported that delayed surgery, hypoproteinemia, three or more comorbidities, and a d-dimer level > 1.59 mg/l were predictors of preoperative DVT, while Kobayashi [15] indicated that female patients, advanced age, prolonged time from injury to admission, prolonged time from injury to surgery, and kidney disease were risk factors for preoperative DVT.

Although a growing number of studies have shown the risks of preoperative DVT, the risk factors for it are still poorly understood. As far as we know, only one meta-analysis [15] has investigated the risk variables for preoperative DVT, and it contains just a few papers. As a result, our meta-analysis evaluated the predictors of preoperative DVT in subgroups of age, body mass index (BMI), ASA class, and fracture type in addition to the overall investigation. It is a hypothesis that many factors are related to preoperative DVT in hip fractures.

## Methods

### Search strategy

We searched for English and with the keywords: "preoperative deep venous thrombosis", "hip fractures", and "risk factors" in the English databases of PubMed, Embase, and Cochrane Library, and the Chinese databases of CNKI and WAN FANG. The date of publication was not restricted, which covered all previously published studies up to January 2022.

### Eligibility criteria

Included articles must meet the following criteria: (1) patients with hip fractures; (2) studies focused on risk factors of preoperative DVT. Studies were excluded if they (1) were abstracts, letters, reviews, or case reports; (2) contained repeated data; (3) did not report outcomes of interest; (4) were patients treated for tumors, infection, or inflammation; or (5) had a history of hip surgeries.

### Data extraction and outcome measures

The data included the general characteristics of each study and the outcomes measured. General characteristics included first author, year of publication, country, the number of patients, and type of article. When the same population was reported in several publications, we retained only the most informative article or complete work to avoid duplication of information. The data were extracted independently by two authors. Any disagreements concerning paper eligibility were resolved by discussion and consensus. We performed a visual inspection of the funnel plot for publication bias. The funnel plot should be asymmetric when there is publication bias and symmetric in the case of no publication bias. We performed Egger and Begg tests to measure the funnel plot asymmetry using a significance level of *p* < 0.10. The trim and fill computation was used to estimate the effect of publication bias. Because of the low heterogeneity of every factor, it implied low sensitivity.

### Statistical analysis

Only continuous outcomes are used in our study, so odd ratios (OR) and 95% confidence intervals (CI) were calculated for those outcomes. A *p* value < 0.05 was judged as statistically significant. Random-effects or fixed-effects models were used depending on the heterogeneity of the studies included. Heterogeneity was analyzed with both the chi-squared test and the *I*^2^ test, where a *p* value of < 0.10 for the chi-squared and *I*^2^ > 50% implied heterogeneity. All statistical analyses were performed using Review Manager version 5.3 (The Cochrane Collaboration, Oxford, UK) and STATA 12.0 (Stata Corporation, College Station, TX, USA).

## Results

### Study identification and selection

**Fig. 1:**
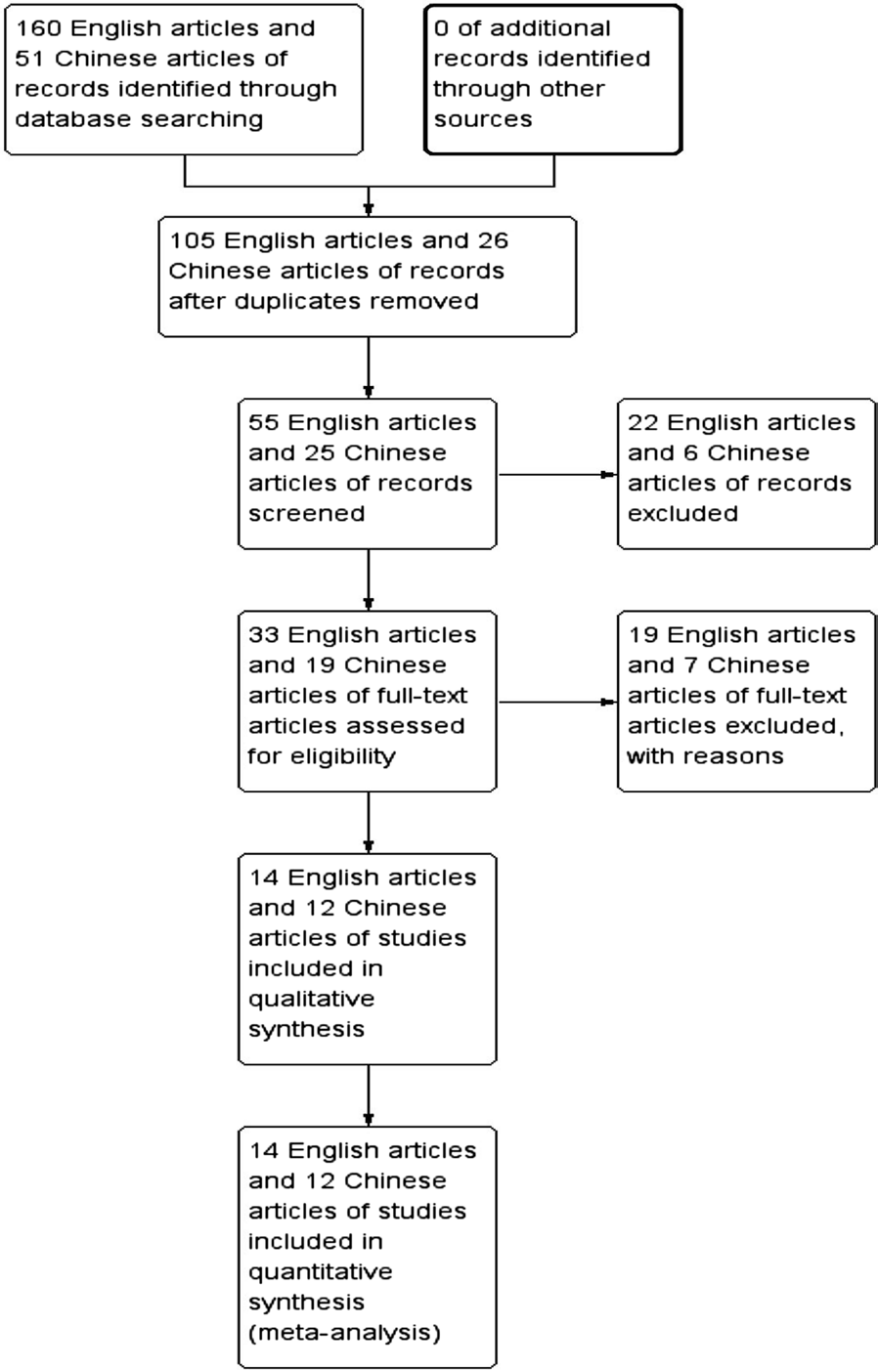
Flow diagram of study selection

Initially, we collected a total of 160 English and 51 Chinese articles through the database search. Of these, 105 English articles and 26 Chinese articles were excluded due to repetition, and 22 English articles and 6 Chinese articles were removed from review on the basis of the titles and abstracts. The remaining 33 English articles and 19 Chinese articles were retrieved for inclusion criteria, and 19 English articles and 7 Chinese articles were excluded. Finally, 14 English articles and 12 Chinese articles that met our inclusion criteria were included in the present meta-analysis. The selection process that was used in this meta-analysis is shown in Fig. 1.

### Baseline characteristics and quality assessment

The main characteristics of the 26 English articles (9823 patients) published before January 2022 and included in the meta-analysis are presented in Table 1.

**Table 1:** Characteristics of included studies

First author	Year	Country	No. of participants		Study type
			DVT	Total	
Shin [10]	2016	Korea	23	208	Respective study
Zhang [11]	2018	China	162	463	Respective study
Zhao [14]	2021	China	155	1515	Respective study
Cho [16]	2015	Korea	4	152	Respective study
Fan [17]	2017	China	35	323	Respective study
Fan [18]	2021	China	164	788	Respective study
Fu [19]	2020	China	73	228	Respective study
Luksameearunothai [20]	2017	Thailand	15	92	Respective study
Niu [21]	2021	China	67	980	Respective study
Song [22]	2016	China	35	119	Respective study
Tan [23]	2016	China	34	386	Respective study
Wang [24]	2018	China	43	392	Respective study
Xia [25]	2018	China	57	301	Respective study
Yu [26]	2020	China	98	334	Respective study
Zeng [27]	2018	China	41	157	Respective study
Zuo [28]	2020	China	116	578	Respective study
Zheng [29]	2021	China	38	272	Respective study
Khan [30]	2019	Pakistan	2	107	Respective study
Xing [31]	2018	China	74	248	Respective study
Zhang [32]	2020	China	84	160	Respective study
Yue [33]	2021	China	85	687	Respective study
Wei [34]	2020	China	58	242	Respective study
Zhang [35]	2020	China	25	105	Respective study
Zhang [36]	2021	China	28	120	Respective study
Guo [37]	2016	China	50	203	Respective study
An [38]	2018	China	61	663	Respective study

Because all studies included were retrospective studies, we used the Newcastle Ottawa Quality Assessment Scale (NOQAS) to assess the quality of each study. This scale for non-randomized case-controlled studies and cohort studies was used to allocate a maximum of nine points for the quality of selection, comparability, exposure, and outcomes for study participants. Twenty of these studies scored eight points, and another six scored seven points. Hence, the quality of each study was relatively high (Table 2).

**Table 2:** The quality assessment according to the Newcastle Ottawa Quality Assessment Scale (NOQAS) of each study

Study	Selection	Comparability	Exposure	Total score
Shin [10]	3	3	2	8
Zhang [11]	3	3	2	8
Zhao [14]	3	2	3	8
Cho [16]	2	3	3	8
Fan [17]	3	2	3	8
Fan [18]	3	2	2	7
Fu [19]	3	3	2	8
Luksameearunothai [20]	3	2	3	8
Niu [21]	3	3	2	8
Song [22]	3	2	3	8
Tan [23]	3	2	3	8
Wang [24]	2	3	2	7
Xia [25]	3	2	3	8
Yu [26]	3	2	2	7
Zeng [27]	3	3	2	8
Zuo [28]	3	2	3	8
Zheng [29]	3	2	3	8
Khan [30]	3	2	2	7
Xing [31]	3	3	2	8
Zhang [32]	3	2	3	8
Yue [33]	2	3	3	8
Wei [34]	3	2	3	8
Zhang [35]	3	2	3	8
Zhang [36]	3	2	3	8
Guo [37]	2	3	2	7
An [38]	2	3	2	7

### Age

**Fig. 2:**
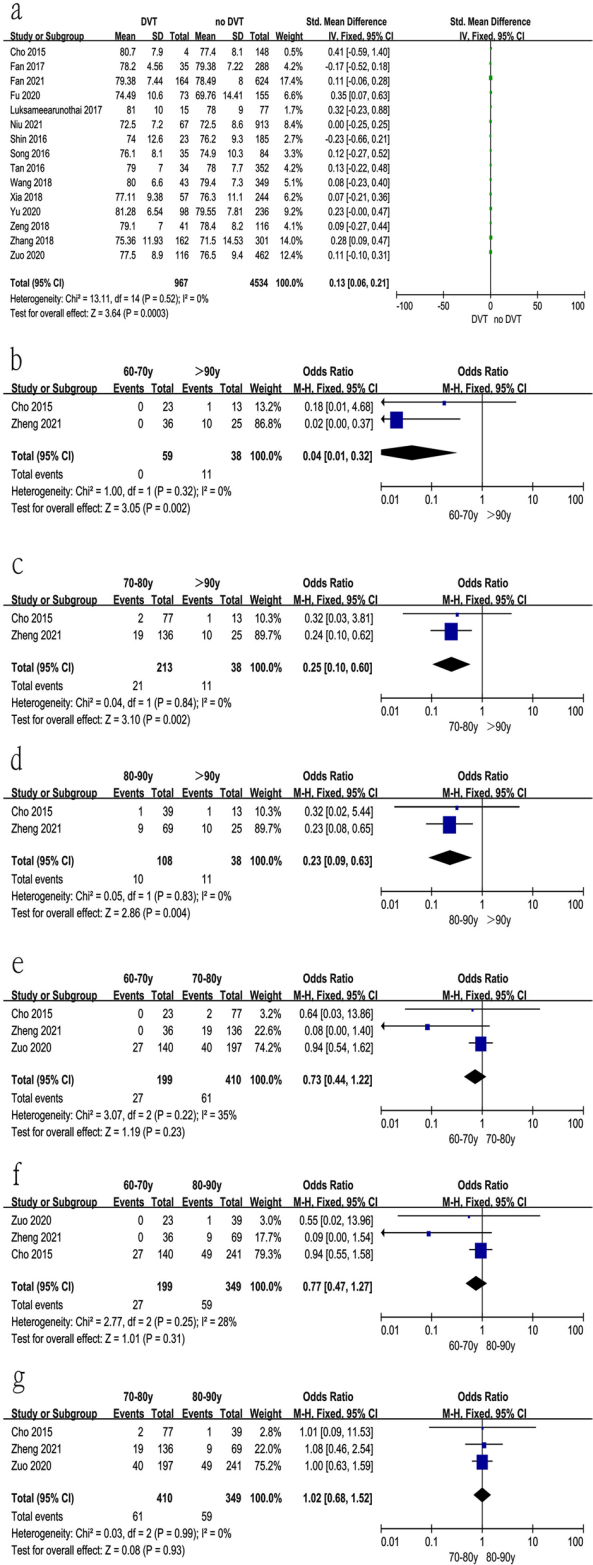
Forest plot showing age in 2 groups. *CI* confidence interval, *df* degrees of freedom, *M-H* Mantel–Haenszel. **a** Relationship between age at surgical time and preoperative DVT; **b** 60–70 years vs > 90 years; **c** 70–80 years vs > 90 years; **d** 80–90 years vs > 90 years; **e** 60–70 years vs 70–80 years; **f** 60–70 years vs 80–90 years; **g** 70–80 years vs 80–90 years

Sixteen studies [10, 11, 16–29] reported the relationship between age at surgical time and preoperative DVT. The test for heterogeneity was not significant and the studies had low heterogeneity (*p* for heterogeneity = 0.52, *I*^2^ = 0%, Fig. 2a and Table 3). In this study, advanced age at surgical time was a risk factor for preoperative DVT [fixed-effects model; *p* = 0.0003, OR = 0.13, 95% CI (0.06, 0.21), Fig. 2a and Table 3]. Furthermore, we analyzed subgroups of age. The results indicated that patients over the age of 90 had an increased risk of preoperative DVT compared with other age groups, as shown in Fig. 2b–d and Table 3. From Fig. 2e–g and Table 3, no significant difference was found among other groups.

**Table 3:** Results

Factors	*I*^*2*^ (%)	*P*	OR	95% CI		*P*
				Lower	Upper	
Age	0	0.52	0.13	0.06	0.21	0.0003
60–70 years vs > 90 years	0	0.32	0.04	0.01	0.32	0.002
70–80 years vs > 90 years	0	0.84	0.25	0.10	0.60	0.002
80–90 years vs > 90 years	0	0.83	0.23	0.09	0.63	0.004
60–70 years vs 70–80 years	35	0.22	0.73	0.44	1.22	0.23
60–70 years vs 80–90 years	28	0.25	0.77	0.47	1.27	0.31
70–80 years vs 80–90 years	0	0.99	1.02	0.68	1.52	0.93
Sex	22	0.16	0.82	0.72	0.92	0.0009
Body mass index	0	0.92	0.07	− 0.03	0.17	0.19
< 18.5 kg/m^2^ vs > 28 kg/m^2^	0	0.84	0.46	0.24	0.90	0.02
24.0–27.9 kg/m^2^ vs > 28 kg/m^2^	0	0.38	0.59	0.39	0.91	0.02
< 25 kg/m^2^ vs > 25 kg/m^2^	0	0.37	1.14	0.55	2.33	0.23
< 18.5 kg/m^2^ vs 18.5–23.9 kg/m^2^	0	0.58	1.00	0.62	1.61	1.00
< 18.5 kg/m^2^ vs 24.0–27.9	0	0.81	0.81	0.50	1.32	0.40
18.5–23.9 kg/m^2^ vs 24.0–27.9 kg/m^2^	0	0.75	0.79	0.62	1.01	0.06
18.5–23.9 kg/m^2^ vs > 28 kg/m^2^	0	0.60	0.71	0.45	1.12	0.14
Type of hip fracture						
Intertrochanteric fracture vs femur neck fracture	0	0.89	1.43	1.20	1.72	< 0.0001
Intertrochanteric fracture vs subtrochanteric fracture	0	0.53	0.28	0.14	0.55	0.0003
Femur neck fracture vs subtrochanteric fracture	0	0.48	0.34	0.19	0.60	0.0002
Time from injury to surgery	0	0.76	2.06	1.40	2.72	< 0.00001
≥ 5 days vs < 5 days	0	0.68	4.54	2.50	8.25	< 0.00001
Time from injury to admission	0	0.49	0.54	0.44	0.65	< 0.00001
Location	0	0.36	1.07	0.72	1.58	0.75
Allergy	0	0.95	0.97	0.67	1.40	0.86
Smoking	0	0.50	1.45	1.11	1.89	0.007
Thrombosis history	0	0.52	5.28	2.85	9.78	< 0.00001
Anti-platelet drug	12	0.32	0.51	0.30	0.85	0.01
Injury side	0	0.84	0.84	0.68	1.03	0.09
Injury mechanism	0	0.54	0.58	0.38	0.87	0.009
ASA class						
ASA III and ASA IV	7	0.34	0.51	0.17	1.53	0.23
ASA I and ASA II	0	0.84	0.39	0.21	0.70	0.002
ASA I and ASA III	0	0.65	0.31	0.17	0.58	0.0002
ASA I and ASA IV	0	0.50	0.13	0.13	0.03	0.49
ASA III and ASA IV	0	0.90	0.21	0.06	0.79	0.02
Hypertension	0	0.55	1.03	0.91	1.16	0.63
Diabetes	0	0.75	1.13	0.90	1.42	0.28
Coronary heart disease	0	0.55	1.25	1.07	1.47	0.006
Cerebrovascular accident	0	0.52	1.09	0.92	1.30	0.32
Cancer	0	0.75	0.84	0.60	1.18	0.33
Dementia	4	0.37	1.72	1.10	2.67	0.02
Liver and kidney Disease	0	0.41	1.91	1.12	3.25	0.02
Liver disease	0	0.53	0.91	0.53	1.57	0.75
Kidney disease	0	0.42	1.26	0.84	1.88	0.26
Pulmonary disease	0	0.57	1.55	1.07	2.33	0.02
Arrhythmia	0	0.61	1.14	0.79	1.63	0.48
Stroke	0	0.66	1.02	0.64	1.64	0.92
Alzheimer’s disease	0	0.44	1.52	0.78	2.97	0.22
Hemoglobin	6	0.38	− 0.32	− 0.43	− 0.21	< 0.00001
Platelet	0	0.56	2.03	− 7.46	11.52	0.68
Activated partial thromboplastin time	0	0.51	− 0.37	− 0.78	0.04	0.08
Prothrombin time	0	0.86	0.15	0.00	0.30	0.05
Fibrinogen	0	0.73	0.20	0.08	0.32	0.0009
D-dimer	0	0.88	− 0.20	− 0.97	0.58	0.62
C-reactive protein	0	0.56	5.95	1.04	10.85	0.01
Albumin	0	0.74	1.42	1.10	1.82	0.006

### Sex

**Fig. 3:**
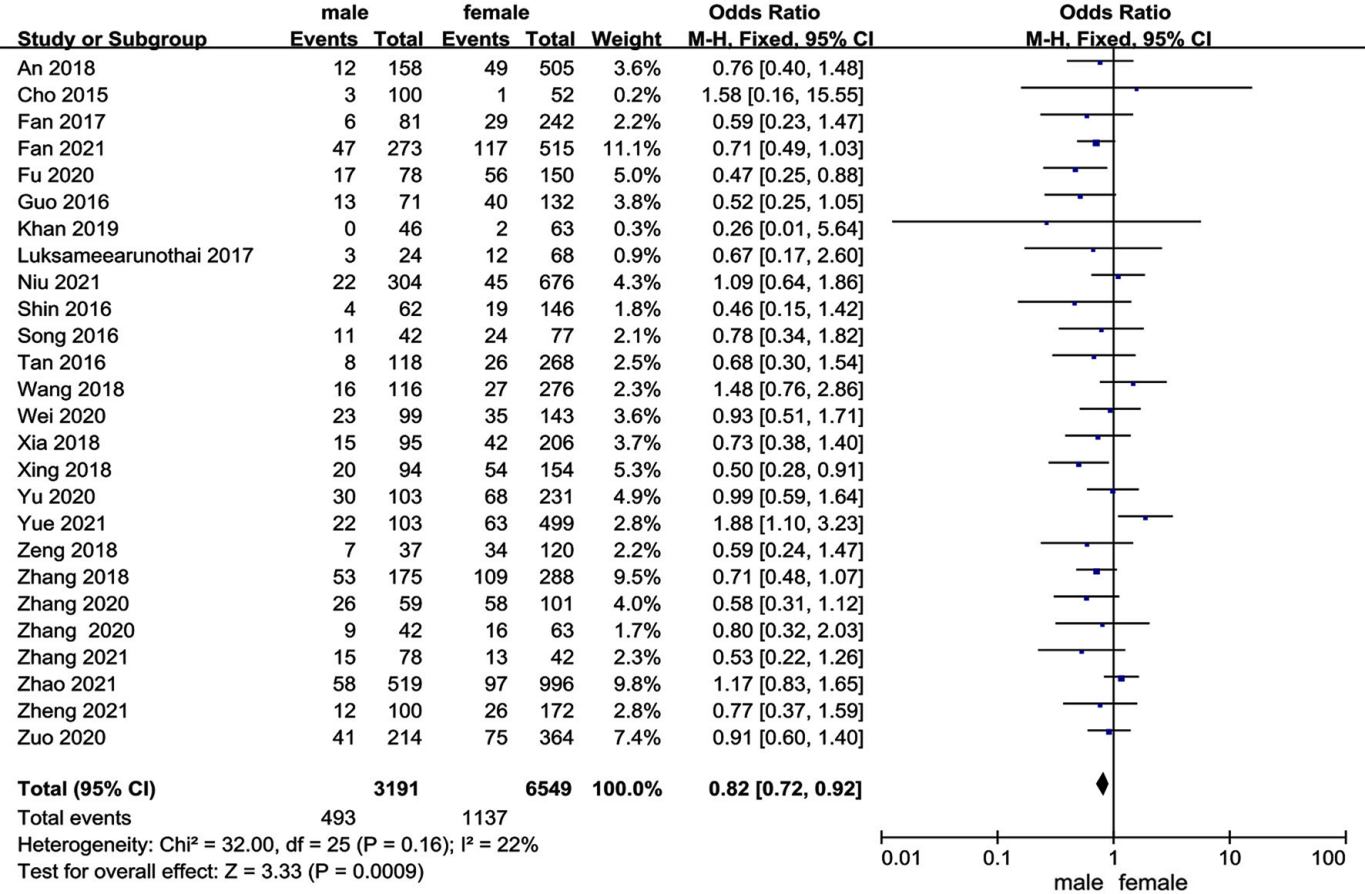
Forest plot showing sex in 2 groups. *CI* confidence interval, *df *degrees of freedom, *M-H* Mantel–Haenszel

Twenty-six studies [10, 11, 14, 16–38] reported the relationship between sex and preoperative DVT. The test for heterogeneity was not significant and the studies had low heterogeneity (*p* for heterogeneity = 0.16, *I*^2^ = 22%). In this study, female patient was a risk factor for preoperative DVT [fixed-effects model; *p* = 0.0009, OR = 0.82, 95% CI (0.72, 0.92)] (Fig. 3 and Table 3).

### Body mass index

**Fig. 4:**
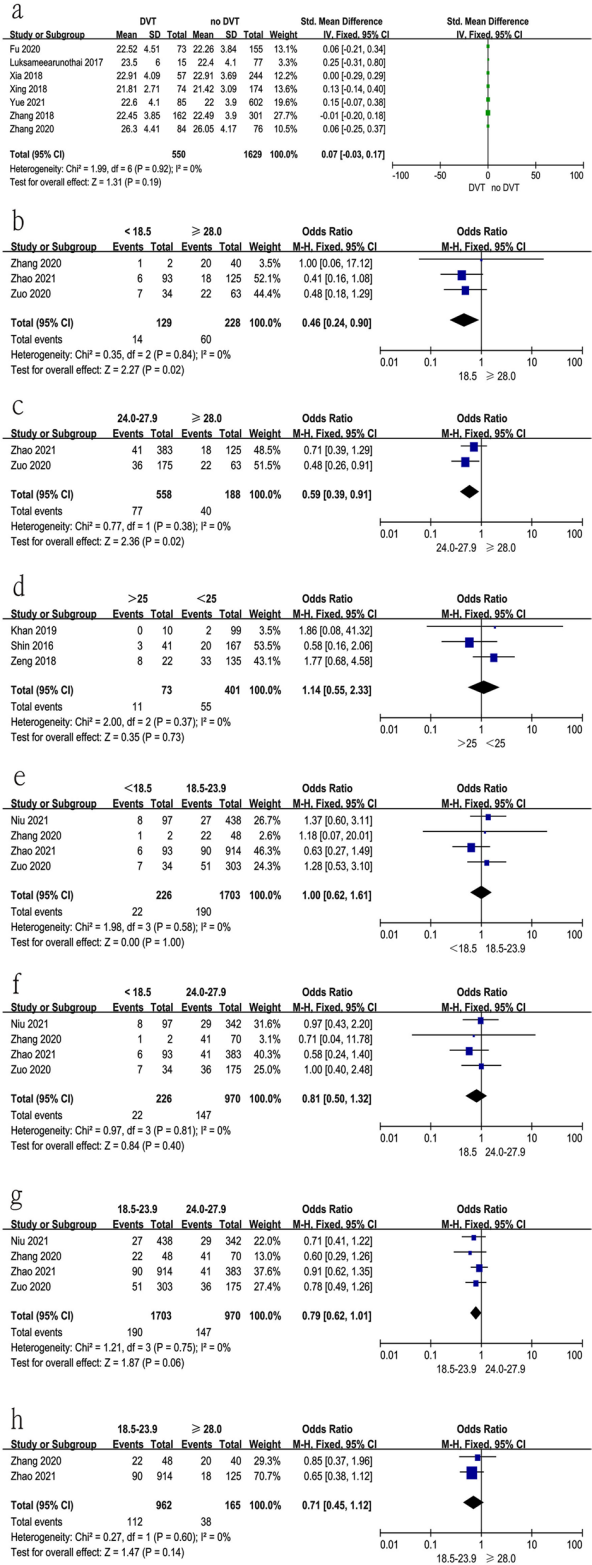
Forest plot showing body mass index in 2 groups. *CI* confidence interval, *df* degrees of freedom, *M-H* Mantel–Haenszel. **a** Relationship between BMI at surgical time and preoperative DVT; **b**  < 18.5 kg/m^2^ vs > 28 kg/m^2^; **c** 24.0–27.9 kg/m^2^ vs > 28 kg/m^2^; **d**  < 25 kg/m^2^ vs > 25 kg/m^2^; **e**  < 18.5 kg/m^2^ vs 18.5–23.9 kg/m^2^; **f**  < 18.5 kg/m^2^ vs 24.0–27.9; **g** 18.5–23.9 kg/m^2^ vs 24.0–27.9 kg/m^2^; **h** 18.5–23.9 kg/m^2^ vs > 28 kg/m^2^

Twelve studies [10–12, 19–21, 25, 27, 30–33] reported the relationship between BMI at surgical time and preoperative DVT. The test for heterogeneity was not significant and the studies had low heterogeneity (*p* for heterogeneity = 0.92, *I*^2^ = 0%, Fig. 4a and Table 3). In this study, BMI at surgical time was not a risk factor for preoperative DVT [fixed-effects model; *p* = 0.19, OR = 0.07, 95% CI (−0.03, 0.17), Fig. 4a and Table 3]. Furthermore, we analyzed subgroups of BMI. The results indicated that patients with more than 28 kg/m^2^ had an increased risk of preoperative DVT compared with the less than 18.5 kg/m^2^ group and 24.0–27.9 kg/m^2^ group, shown in Fig. 4b, c and Table 3. From Fig. 4d–h and Table 3, there was no significant difference among other groups.

### Type of hip fracture

**Fig. 5:**
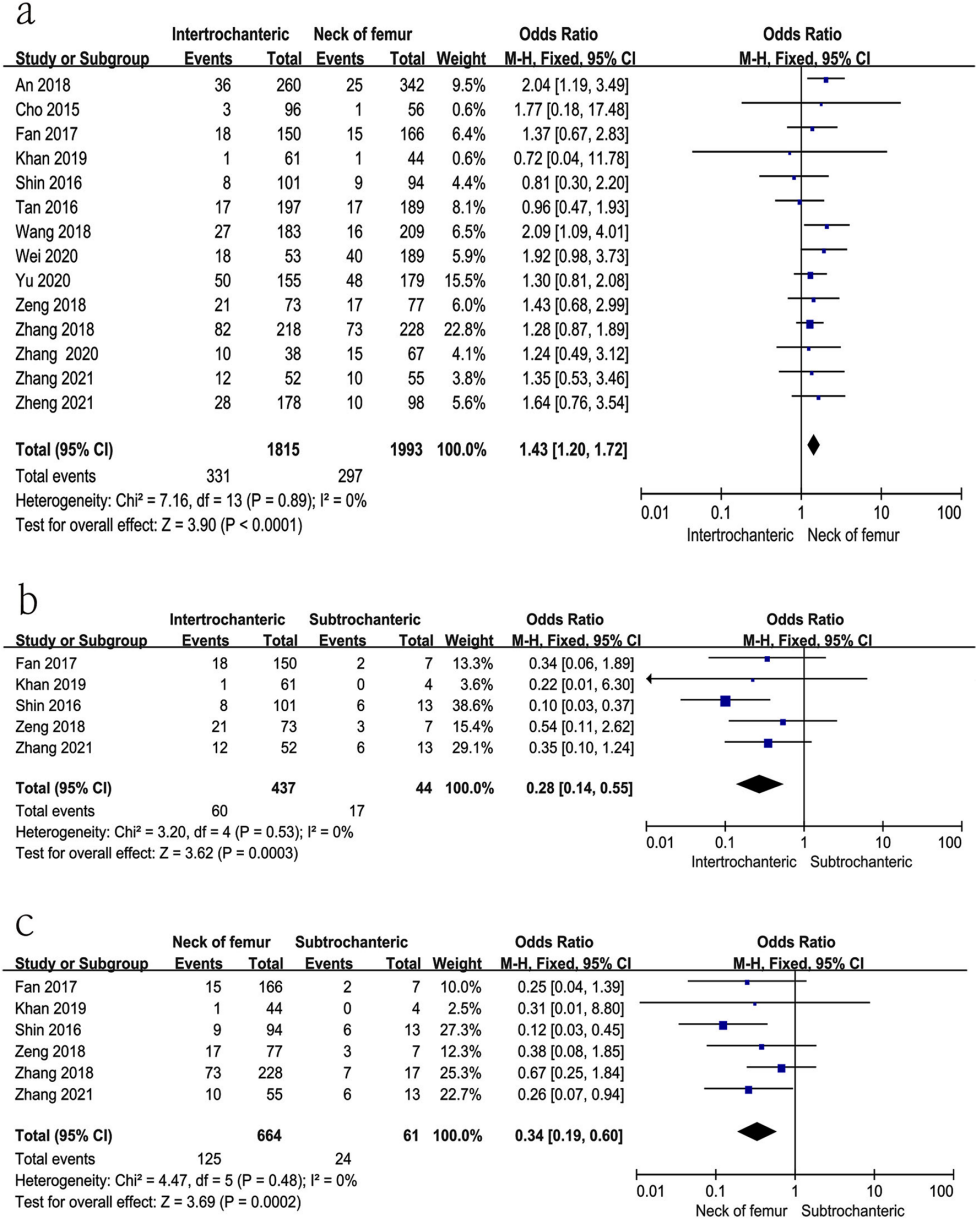
Forest plot showing type of fracture in 2 groups. *CI* confidence interval, *df* degrees of freedom, *M-H* Mantel–Haenszel. **a** Intertrochanteric fracture vs femur neck fracture; **b** intertrochanteric fracture vs subtrochanteric fracture; **c** femur neck fracture vs subtrochanteric fracture

Fourteen studies [10, 11, 16, 17, 23, 24, 26, 27, 29, 30, 34–36, 38] reported the relationship between type of hip fracture and preoperative DVT. Intertrochanteric fracture vs femur neck fracture: p for heterogeneity = 0.89, *I*^2^ = 0%; Intertrochanteric fracture vs subtrochanteric fracture: *p* for heterogeneity = 0.53, *I*^2^ = 0%; Femur neck fracture vs subtrochanteric fracture: *p* for heterogeneity = 0.48, *I*^2^ = 0%). In this study, patients with subtrochanteric fracture had the highest rate of preoperative DVT, while femur neck fracture had the lowest rate [intertrochanteric fracture vs femur neck fracture, fixed-effects model; *p* < 0.0001, OR = 1.43, 95% CI (1.20, 1.72); intertrochanteric fracture vs subtrochanteric fracture, fixed-effects model; *p* = 0.0003, OR = 0.28, 95% CI (0.14, 0.55); femur neck fracture vs subtrochanteric fracture, fixed-effects model; *p* = 0.0002, OR = 0.34, 95% CI (0.19, 0.60)] (Fig. 5 and Table 3).

### Time from injury to surgery

**Fig. 6:**
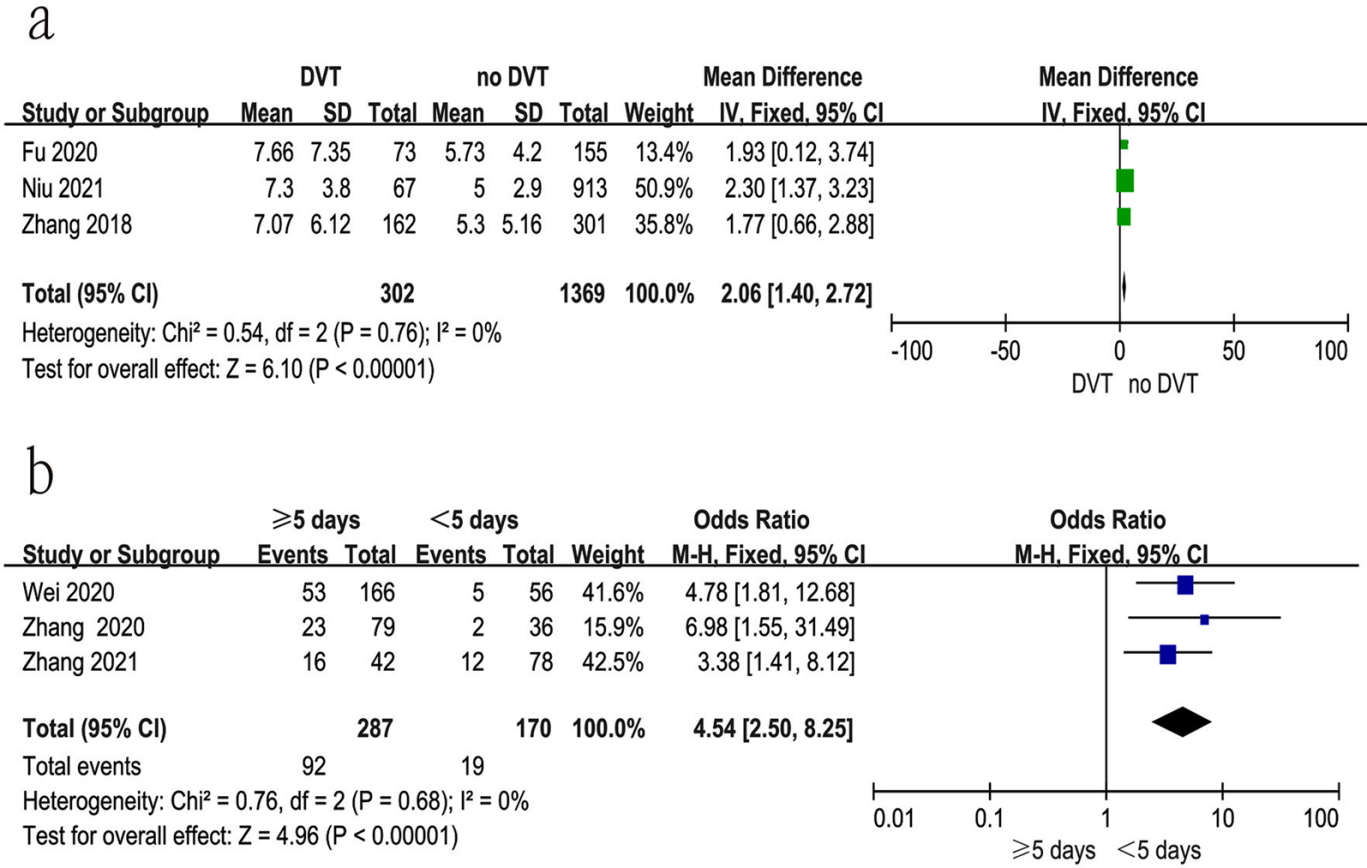
Forest plot showing prolonged time from injury to admission in 2 groups. *CI* confidence interval, *df* degrees of freedom, *M-H* Mantel–Haenszel. **a** Prolonged time from injury to admission; **b**  ≥ 5 days vs < 5 days

Six studies [11, 19, 21, 34–36] reported the relationship between time from injury to surgery and preoperative DVT. The test for heterogeneity was not significant and the studies had low heterogeneity (*p* for heterogeneity = 0.76, *I*^2^ = 0%). In this study, prolonged time from injury to admission was a risk factor for preoperative DVT [fixed-effects model; *p* < 0.00001, OR = 2.06, 95% CI (1.40, 2.72)]. Moreover, we also explored whether 5 days as a cut-off time affected the rate of preoperative DVT. The test for heterogeneity was not significant and the studies had low heterogeneity (*p* for heterogeneity = 0.68, *I*^2^ = 0%). In this study, ≥ 5 days from injury to admission had a significantly higher rate of preoperative DVT compared with < 5 days [fixed-effects model; *p* < 0.00001, OR = 4.54, 95% CI (2.50, 8.25)] (Fig. 6 and Table 3).

### Time from injury to admission

**Fig. 7:**
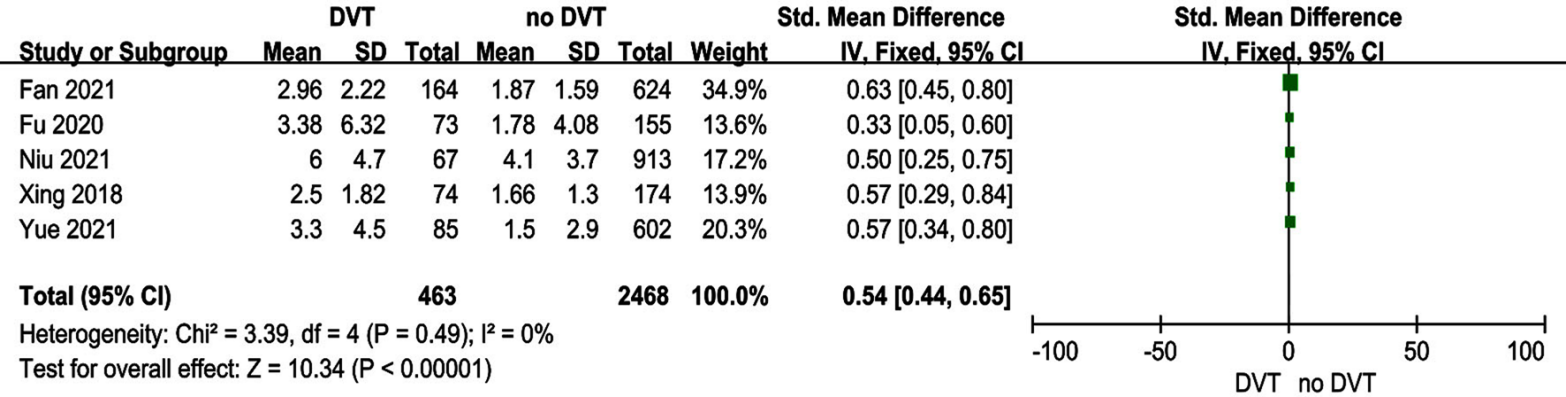
Forest plot showing time from injury to admission in 2 groups. *CI* confidence interval, *df* degrees of freedom, *M-H* Mantel–Haenszel

Five studies [18, 19, 21, 31, 33] reported the relationship between time from injury to admission and preoperative DVT. The test for heterogeneity was not significant and the studies had low heterogeneity (*p *for heterogeneity = 0.49, *I*^2^ = 0%). In this study, prolonged time from injury to admission was a risk factor for preoperative DVT [fixed-effects model; *p* < 0.00001, OR = 0.54, 95% CI (0.44, 0.65)] (Fig. 7 and Table 3).

### Location

**Fig. 8:**
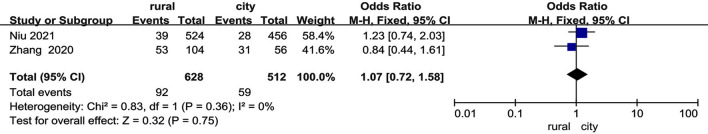
Forest plot showing the relationship between the location of living place and preoperative DVT in 2 groups. *CI* confidence interval, *df* degrees of freedom, *M-H* Mantel–Haenszel

Two studies [21, 32] reported the relationship between the location of living place (rural or city) and preoperative DVT. The test for heterogeneity was not significant and the studies had low heterogeneity (*p* for heterogeneity = 0.36, *I*^2^ = 0%). In this study, city location was not a risk factor for preoperative DVT [fixed-effects model; *p* = 0.75, OR = 1.07, 95% CI (0.72, 1.58)] (Fig. 8 and Table 3).

### Allergy

**Fig. 9:**
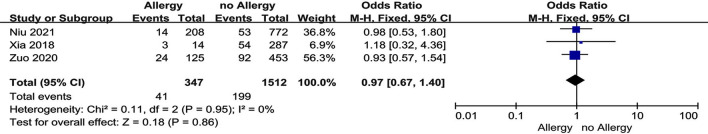
Forest plot showing a history of allergy in 2 groups. *CI* confidence interval, *df* degrees of freedom, *M-H* Mantel–Haenszel

Three studies [21, 25, 28] reported the relationship between patients with a history of allergy and preoperative DVT. The test for heterogeneity was not significant and the studies had low heterogeneity (*p* for heterogeneity = 0.95, I^2^ = 0%). In this study, a history of allergy was not a risk factor for preoperative DVT [fixed-effects model; *p* = 0.86, OR = 0.97, 95% CI (0.67, 1.40)] (Fig. 9 and Table 3).

### Smoking

**Fig. 10:**
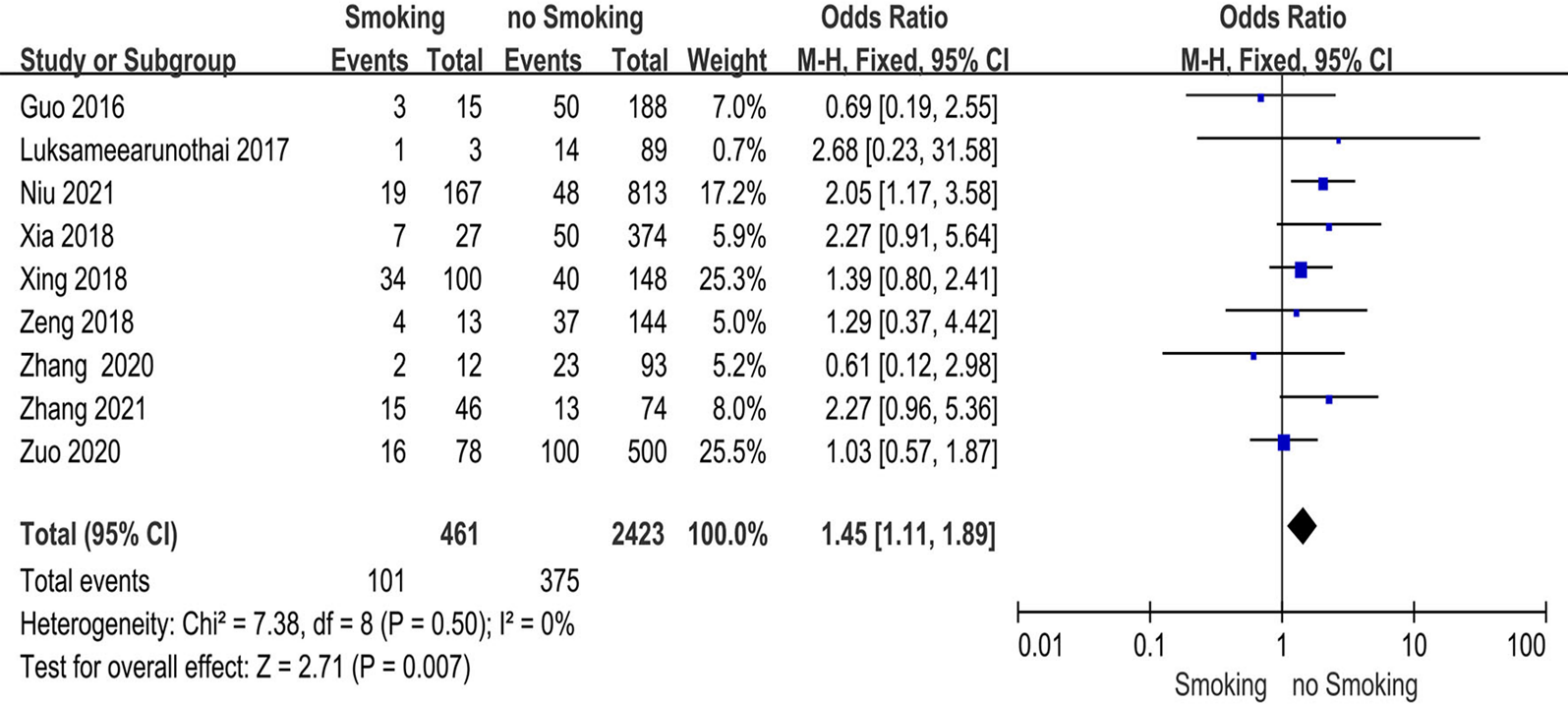
Forest plot showing a history of smoking in 2 groups. *CI* confidence interval, *df* degrees of freedom, *M-H* Mantel–Haenszel

Nine studies [20, 21, 25, 27, 28, 31, 35–37] reported the relationship between patients with a history of smoking and preoperative DVT. The test for heterogeneity was not significant and the studies had low heterogeneity (*p* for heterogeneity = 0.50, *I*^2^ = 0%). In this study, a history of smoking was a risk factor for preoperative DVT [fixed-effects model; *p* = 0.007, OR = 1.45, 95% CI (1.11, 1.89)] (Fig. 10 and Table 3).

### Thrombosis history

**Fig. 11:**
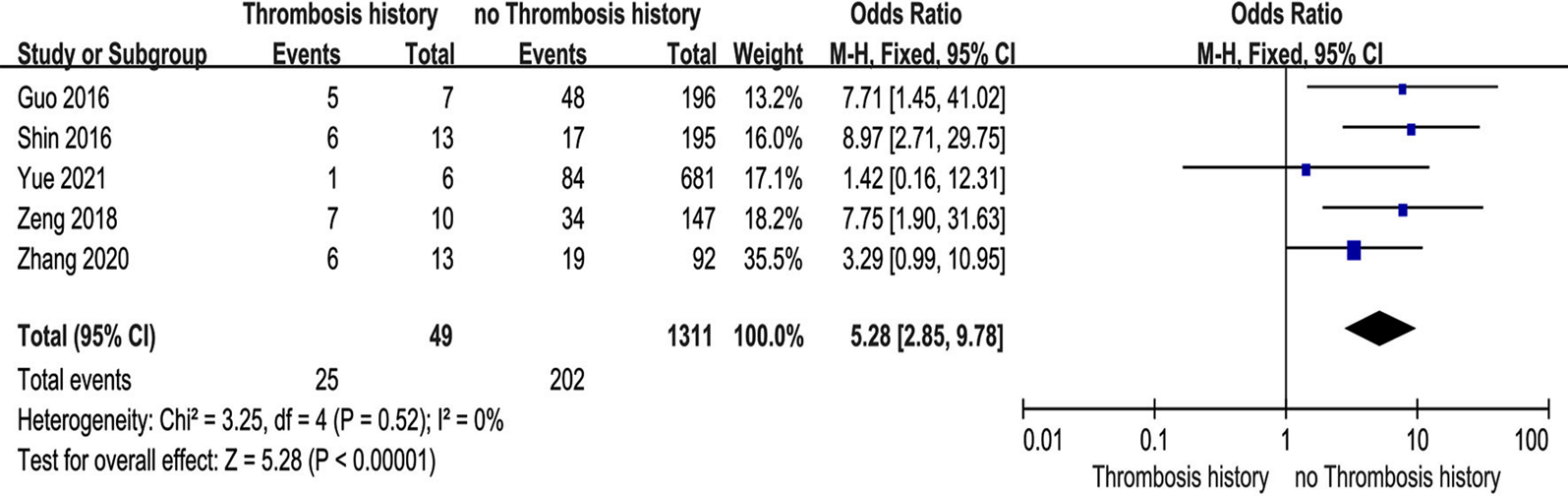
Forest plot showing the relationship between patients with a history of thrombosis and preoperative DVT in 2 groups. *CI* confidence interval, *df* degrees of freedom, *M-H* Mantel–Haenszel

Five studies [10, 27, 33, 35, 37] reported the relationship between patients with a history of thrombosis and preoperative DVT. The test for heterogeneity was not significant and the studies had low heterogeneity (*p* for heterogeneity = 0.52, *I*^2^ = 0%). In this study, a history of thrombosis was a risk factor for preoperative DVT [fixed-effects model; *p* < 0.00001, OR = 5.28, 95% CI (2.85, 9.78)] (Fig. 11 and Table 3).

### Anti-platelet drug

**Fig. 12:**
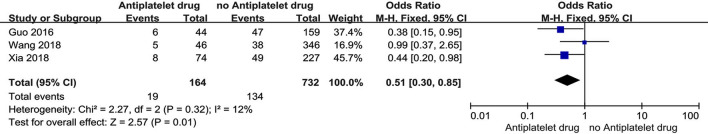
Forest plot showing the relationship between anti-platelet drug and preoperative DVT in 2 groups. *CI* interval, *df* degrees of freedom, *M-H* Mantel–Haenszel

Three studies [24, 25, 37] reported the relationship between the anti-platelet drug and preoperative DVT. The test for heterogeneity was not significant and the studies had low heterogeneity (*p* for heterogeneity = 0.32, *I*^2^ = 12%). In this study, not taking an anti-platelet drug was a risk factor for preoperative DVT [fixed-effects model; *p* = 0.01, OR = 0.51, 95% CI (0.30, 0.85)] (Fig. 12 and Table 3).

### Injury side

**Fig. 13:**
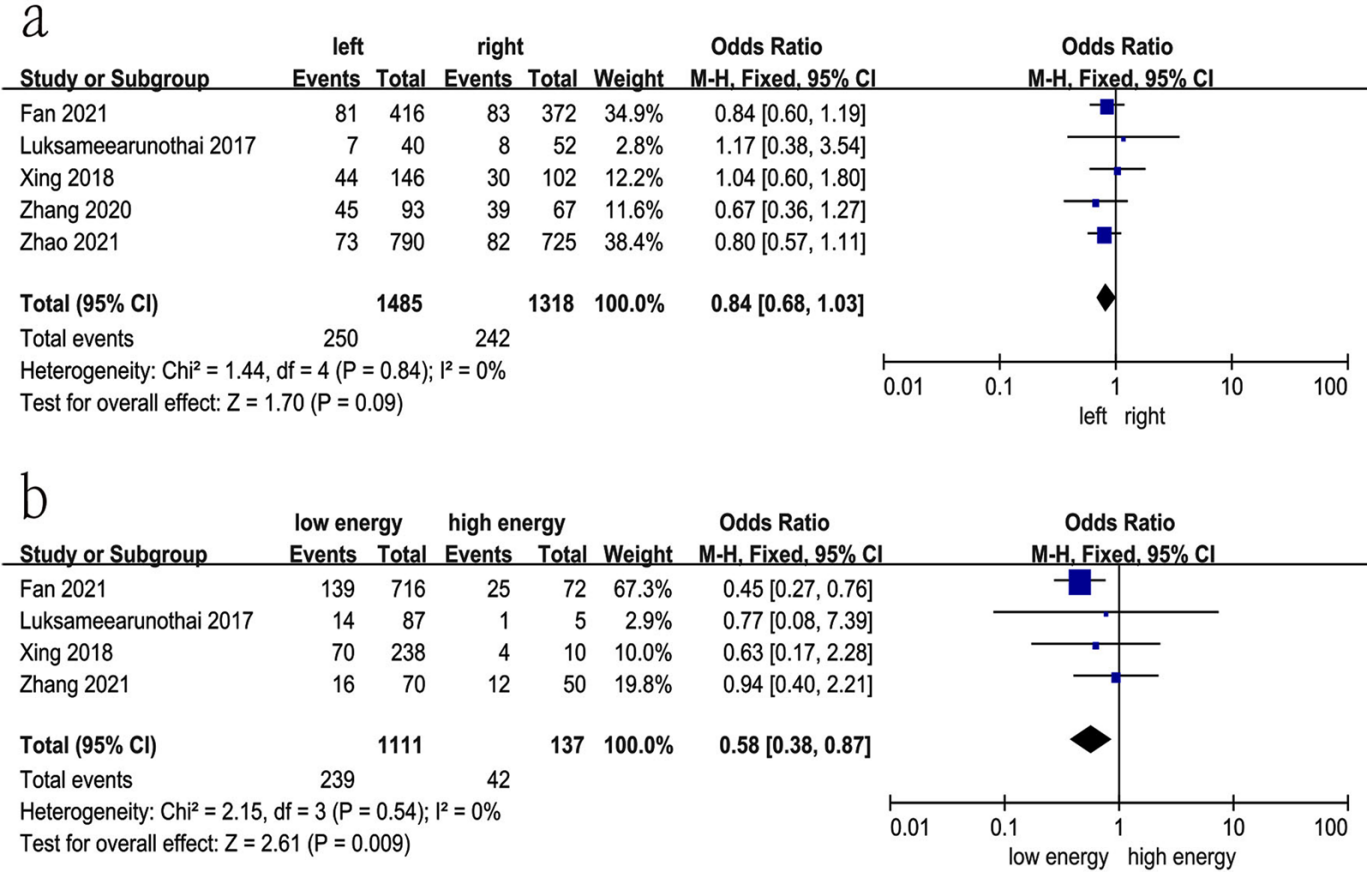
Forest plot showing the relationship between injury side and mechanism and preoperative DVT in 2 groups. *CI* confidence interval, *df* degrees of freedom, *M-H* Mantel–Haenszel. **a** Injury side; **b** injury mechanism

Five studies [14, 18, 20, 22, 31] reported the relationship between injury side and preoperative DVT. The test for heterogeneity was not significant and the studies had low heterogeneity (*p* for heterogeneity = 0.84, *I*^2^ = 0%). In this study, injury side was not a risk factor for preoperative DVT [fixed-effects model; *p* = 0.09, OR = 0.84, 95% CI (0.68, 1.03)] (Fig. 13a and Table 3).

### Injury mechanism

Four studies [18, 22, 31, 36] reported the relationship between injury mechanism and preoperative DVT. The test for heterogeneity was not significant and the studies had low heterogeneity (*p* for heterogeneity = 0.54, *I*^2^ = 0%). In this study, high-energy injury was a risk factor for preoperative DVT [fixed-effects model; *p* = 0.009, OR = 0.58, 95% CI (0.38, 0.87)] (Fig. 13b and Table 3).

### ASA class

**Fig. 14:**
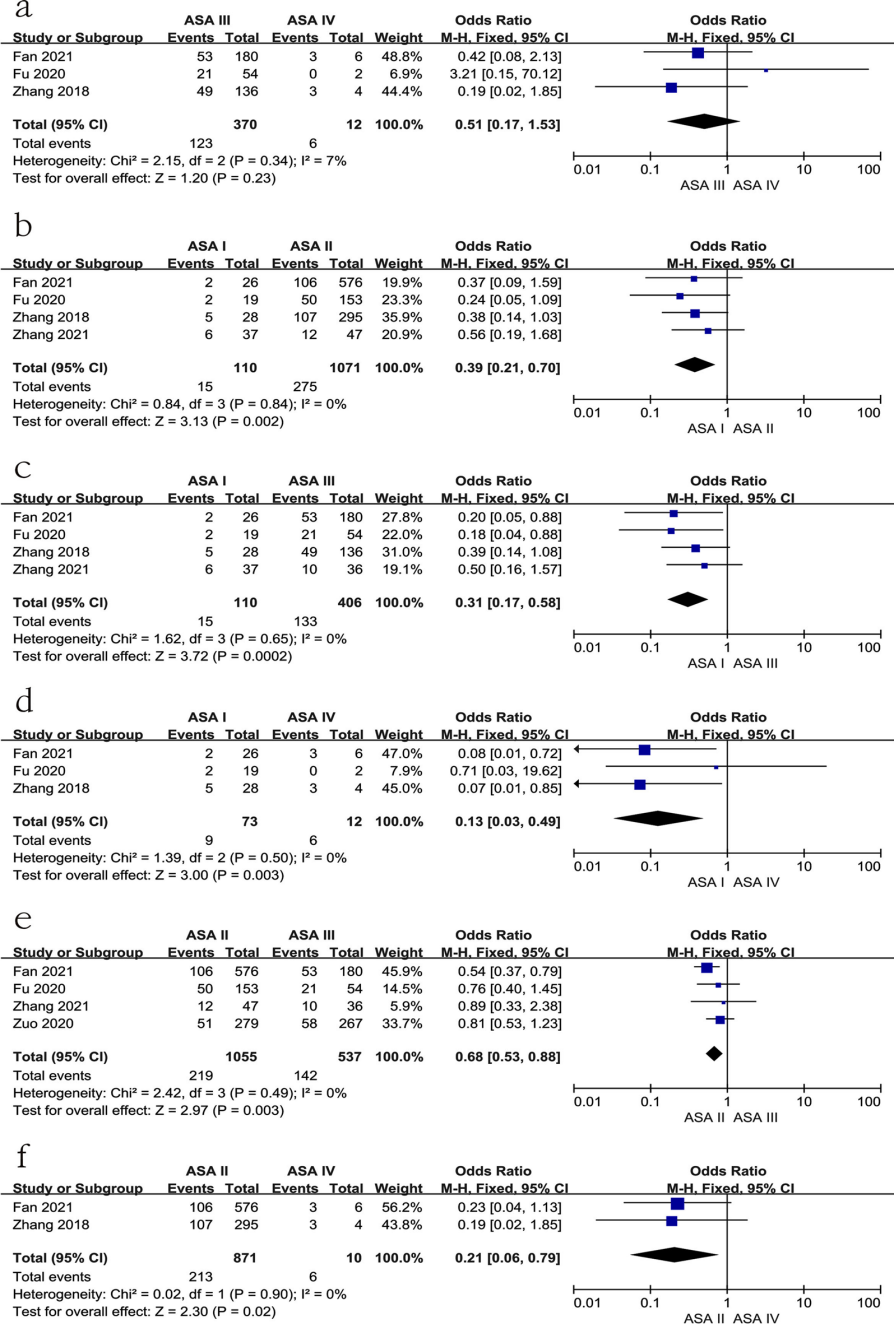
Forest plot showing ASA class in 2 groups. *CI* confidence interval, *df* degrees of freedom, *M-H* Mantel–Haenszel. **a** ASA III and ASA IV; **b** ASA I and ASA II; **c** ASA I and ASA III; **d** ASA I and ASA IV; **e** ASA II and ASA III; **f** ASA III and ASA IV

Five studies [11, 18, 19, 28, 36] reported the relationship between subgroups of ASA class and preoperative DVT. No significant difference was found between ASA III and ASA IV [*p* for heterogeneity = 0.34, *I*^2^ = 7%, *p* = 0.23, OR = 0.51, 95% CI (0.17, 1.53), Fig. 14a and Table 3]. Furthermore, there were significant differences among other groups, as shown in Fig. 14b–f and Table 3).

### Medical history

**Fig. 15:**
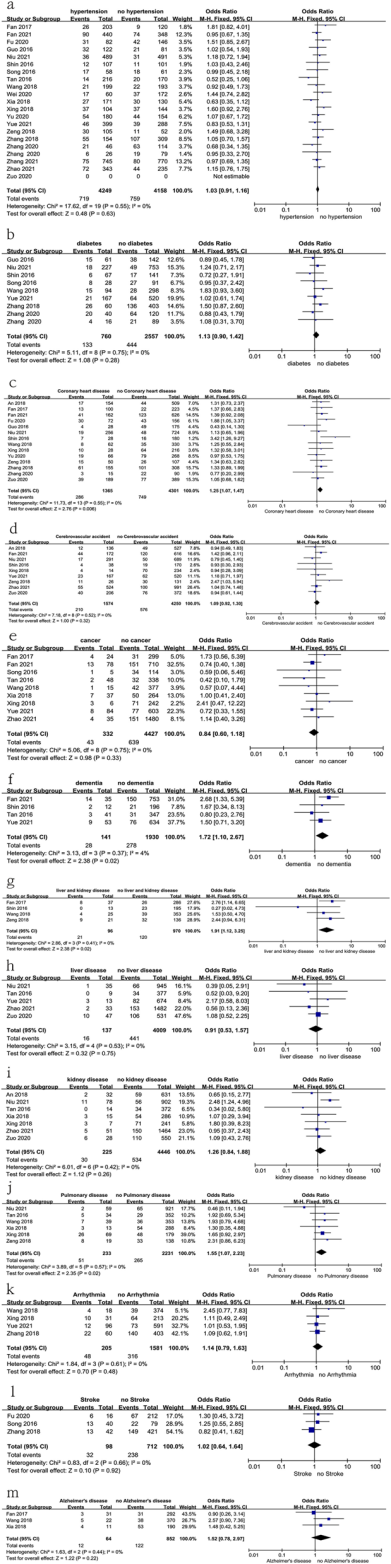
Forest plot showing the relationship between medical history and preoperative DVT in 2 groups. *CI* confidence interval, *df* degrees of freedom, *M-H* Mantel–Haenszel. **a** Hypertension; **b** diabetes; **c** coronary heart disease; **d** cerebrovascular accident; **e** cancer; **f** dementia; **g** liver and kidney disease; **h** liver disease; **i** kidney disease; **j** pulmonary disease; **k** arrhythmia; **l** stroke; **m** Alzheimer’s disease

Twenty-two studies [10, 11, 14, 17–19, 21–28, 31–38] reported the relationship between patients with medical history and preoperative DVT. In this study, there was no significant difference in patients with a history of hypertension, diabetes, cerebrovascular accident, cancer, liver disease, kidney disease, arrhythmia, stroke, or Alzheimer’s disease, as shown in Fig. 15 and Table 3. However, a history of coronary heart disease, dementia, liver and kidney disease, or pulmonary disease were found to be risk factors for preoperative DVT, as shown in Fig. 15 and Table 3.

### Laboratory tests

**Fig. 16:**
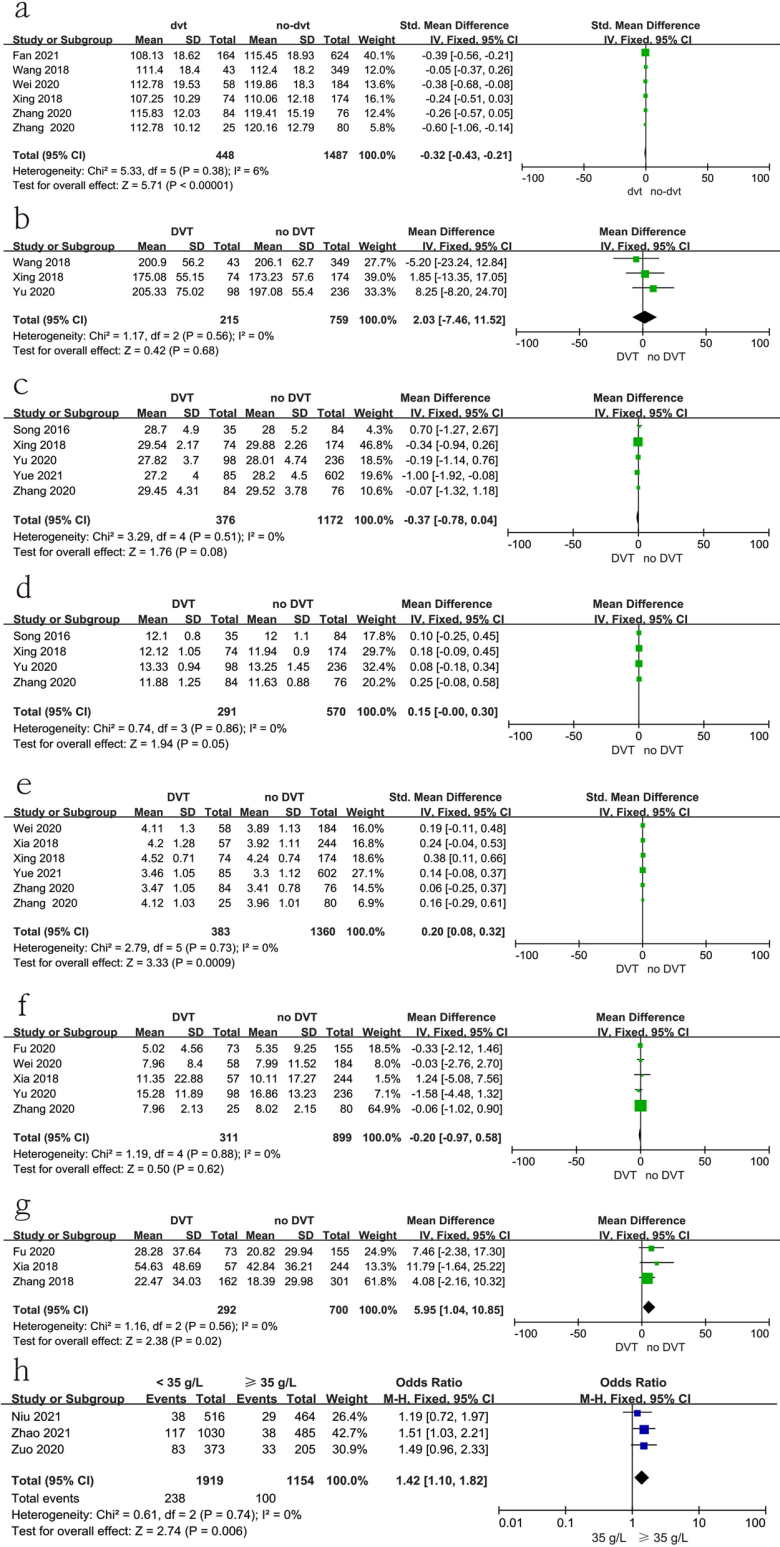
Forest plot showing the relationship between laboratory test and preoperative DVT in 2 groups. *CI* confidence interval, *df* degrees of freedom, *M-H* Mantel–Haenszel. **a** Hemoglobin; **b** platelet; **c** activated partial thromboplastin time (APTT); **d** activated prothrombin time (PT); **e** fibrinogen; **f** D-dimer; **g** C-reactive protein (CRP); **h** albumin (< 35 g/l vs ≥ 35 g/l)

Fifteen studies [11, 14, 18, 19, 21, 22, 24–26, 28, 31–35] reported the relationship between laboratory tests at admission and preoperative DVT. In this study, a low level of hemoglobin; or a high level of CRP and fibrinogen, or albumin (< 35 g/l) were risk factors for preoperative DVT, as shown in Fig. 16 and Table 3. However, there was no significant difference in the level of platelets, activated partial thromboplastin time (APTT), prothrombin time (PT), or D-dimer in the two groups, as shown in Fig. 16 and Table 3.

### Publication bias

After detection of publication bias by STATA 12.0, there was no publication bias found for any included studies (all *P* > 0.05).

## Discussion

Preoperative DVT is one of the most common complications after hip fractures because of immobilization and medical problems of patients. Early prevention of preoperative DVT was beneficial in shortening the time from injury to surgery and lowering the incidence of postoperative complications [12]. Medical problems and prolonged time from injury to admission have recently been linked to an increased risk of preoperative DVT [10, 11, 14, 16–38], but other factors, such as advanced age or D-dimer level, remain controversial. In our meta-analysis, the rate of preoperative DVT was 16.6% (1627 of 9823 patients). Additionally, advanced age, female patients, high BMI (> 28 kg/m^2^), high-energy injury, prolonged time from injury to admission, prolonged time from injury to surgery, type of hip fracture, low level of hemoglobin, a history of coronary heart disease, dementia, high ASA class (III and IV), liver and kidney diseases, dementia, pulmonary disease, smoking and thrombosis, high level of fibrinogen and CRP, patients without anti-platelet drug, and patients with < 35 g/l albumin were risk factors for preoperative DVT.

Advanced age was considered to be an independent predictor of preoperative DVT by prior articles [13, 29, 31, 32], which was consistent with our findings. However, some articles [10, 18, 25] obtained the opposite results. Compared with those aged 49 years, Yeol et al. [39] discovered a fivefold increase and a tenfold increase in the risk of DVT in patients aged 50–69 years and those older than 70 years, respectively. Dong [40] found that being older than 60 years old was an independently related risk for DVT. To further assess the cut-off value of age above which the risk of DVT increases significantly, we analyzed age subgroups. Patients over the age of 90 had a significantly higher rate of preoperative DVT than any other age group, which was linked to a prothrombotic state and decreased vascular function due to aging. Another debated factor was the gender of patients. Our findings indicated that being female was related to preoperative DVT, which may be explained by the genetic differences and hormonal changes following menopause and its associated complications [41, 42]. Regarding the BMI of patients, no significant difference was found between the DVT group and the non-DVT group. Whereas, in the subgroups of BMI, patients with more than 28 kg/m^2^ were found to have a higher rate of preoperative DVT compared with the less than 18.5 kg/m^2^ group and the 24.0–27.9 kg/m^2^ group. Increased BMI was associated with venous thromboembolism, with multiple mechanisms and pathways contributing to this effect [42].

In terms of laboratory tests, D-dimer, which is affected by a variety of variables such as inflammation, age, surgery, hospitalization, COPD, and other acute disorders [43–46], is commonly used to detect DVT. In our research, no significant difference in D-dimer levels was identified between the DVT and non-DVT groups, which contradicted prior findings [11, 22, 31]. Furthermore, there was debate concerning the D-dimer cut-off value for the diagnosis of DVT in older patients, particularly in HF patients [14, 31]. The use of age in conjunction with D-dimer as a crucial value enhanced the prediction accuracy for DVT development and should be encouraged. In addition to D-dimer, a high level of fibrinogen was also recognized as a risk factor for preoperative DVT in the present study, which may be associated with a hypercoagulable state due to increased fibrin network density, blood viscosity, and the resistance of clots to fibrinolysis caused by elevated fibrinogen [47–49]. CRP, as an inflammatory protein biomarker, plays a role in the inflammatory process [50]. Zhang [32] discovered that CRP levels greater than 11 mg/l increase the probability of preoperative DVT by 4.16 times. Studies have documented that CRP levels are elevated in DVT [51, 52], and this was supported by our results. CRP has been shown to have a positive relationship with D-dimer, which might be related to D-dimer and other fibrin breakdown products’ propensity to upregulate interleukin-6 production, which stimulates CRP synthesis [53]. Moreover, patients with albumin < 35 g/l were associated with an excess risk of DVT in this study, which was consistent with recent studies [54, 55], and might be explained by the hyperfibrinogenemia and platelet aggregability triggered by hypoalbuminemia [56]. Furthermore, preoperative anemia has been demonstrated to be a risk factor for preoperative DVT in hip fracture patients [54], In the present study, patients with preoperative DVT tended to have a lower level of hemoglobin (Additional file 1).

The ASA classification system is a routinely used rating scale for in-patients’ physical condition, anesthetic tolerance, and surgical tolerance at the time of admission. In this investigation, ASA II-IV corresponded with a significantly higher incidence of preoperative DVT than ASA I, which was consistent with a nationwide study [57]. In terms of medical history, the results showed that a history of coronary heart disease, dementia, liver and renal illness, dementia, pulmonary disease, smoking, and thrombosis increased the risk of preoperative DVT, which was consistent with prior research [16–38]. The cause of the link might be hypercoagulability or vascular dysfunction in individuals prior to fracture.

In the present study, the mean time from injury to admission and surgery was significantly longer in the DVT group than in the non-DVT group. Three possible reasons may explain this. First, prolonged immobilization could result in venous congestion; second, vascular injury caused by fracture activated the coagulation system; third, the fracture was frequently coupled with dominant and hidden blood loss, especially hidden blood loss for intertrochanteric fractures. Therefore, earlier admission was necessary for intertrochanteric fractures.

Furthermore, different from some previous studies [11, 16, 30], we found the development of DVT after hip fracture was influenced by the type of fracture. In our study, patients with subtrochanteric fractures had the highest rate of preoperative DVT, while those with femur neck fractures had the lowest rate. This may be associated with severe vascular injury and the complexity of subtrochanteric fractures commonly caused by high-energy injuries.

There were several limitations to this study. First, there was no RCT article focused on this topic. We needed an RCT to perform a further study. Second, the statistical power could be improved in the future by including more studies. Due to the small number of included studies, a subgroup of some predictors could not be analyzed. However, as far as we know, there was only one meta-analysis including nine articles to explore the risk factors of preoperative DVT after hip fracture. We collected more articles and we analyzed subgroups of age, BMI, ASA class, and fracture type. Third, the search strategy was restricted to articles published in English and Chinese. Articles with potentially high-quality data that were published in other languages were not included because of anticipated difficulties in obtaining accurate medical translations. Fourth, all the included articles originated from Asian countries, which may influence the accuracy of the results. However, all the included articles were of high quality, and all the predictors had low heterogeneity.

In summary, advanced age, female patients, some medical history, and laboratory index were found to be associated with preoperative DVT after hip fracture. We hope our findings can be a guide for orthopedic surgeons. We still need more studies to be included in future studies.

## Supplementary Information

**Additional file 1.** PRISMA 2009 flow diagram.

## Data Availability

Yes.

## Abbreviations

DVT: Deep venous thrombosis
HF: Hip fractures
APTT: Activated partial thromboplastin time
PT: Prothrombin time
CRP: C-reactive protein
BMI: Body mass index
