# Supplementary material for: Risk factors for preoperative deep venous thrombosis in hip fracture patients: a meta-analysis
Source: J Orthop Traumatol. 2022 Apr 7;23:19. doi: 10.1186/s10195-022-00639-6 (PMC8991371; doi:10.1186/s10195-022-00639-6)
Supplement: Supplementary file 1 — Additional file 1. PRISMA 2009 flow diagram. [file 10195_2022_639_MOESM1_ESM.doc]

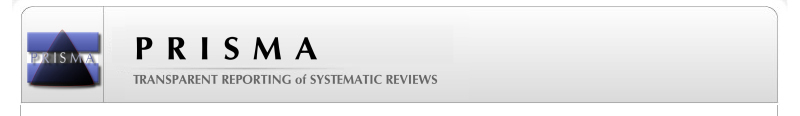
**PRISMA 2009 Flow Diagram**

**Screening**

**Included**

**Eligibility**

**Identification**

Records identified through database searching
(n =211 )

Additional records identified through other sources
(n = 0 )

Records after duplicates removed
(n = 131 )

Records screened
(n = 80 )

Records excluded
(n = 28 )

Full-text articles assessed for eligibility
(n = 52 )

Full-text articles excluded, with reasons
(n = 26 )

Studies included in qualitative synthesis
(n =26)

Studies included in quantitative synthesis (meta-analysis)
(n =26 )
